# Understanding viral blips in people with HIV on antiretroviral therapy: a nationwide Swedish cohort study of 4,210 individuals

**DOI:** 10.1186/s12879-026-14654-z

**Published:** 2026-10-16

**Authors:** Erik Sörstedt, Staffan Nilsson, Anders Sönnerborg, Christina Carlander, Carl Johan Treutiger, Fredrik Månsson, Lena Änghagen, Helena Berggren, Magnus Gisslén, Aylin Yilmaz

**Affiliations:** 1 Department of Infectious Diseases, Sahlgrenska Academy, Institute of Biomedicine, University of Gothenburg, Gothenburg, SE-416 45 Sweden; 2 Department of Infectious Diseases, Region Västra Götaland, Sahlgrenska University Hospital, Gothenburg, Sweden; 3 Department of Laboratory Medicine, Institute of Biomedicine, Sahlgrenska Academy, University of Gothenburg, Gothenburg, Sweden; 4 Department of Medicine Huddinge, Division of Infectious Diseases, Karolinska Institutet, Stockholm, Sweden; 5 Department of Infectious Diseases, Karolinska University Hospital, Stockholm, Sweden; 6 Department of Infectious Diseases/Venhälsan, South General Hospital, Stockholm, Sweden; 7 Department of Translational Medicine, Infectious Diseases Unit, Lund University, Malmö, Sweden; 8 University of Linköping, Linköping, Sweden; 9 Department of Infectious Diseases, Linköping University Hospital, Linköping, Sweden; 10 Department of Medical Sciences, Section of Infectious Diseases, Uppsala University, Uppsala, Sweden

**Keywords:** HIV-1, Viral blips, Virological failure, Transient viremia, Integrase inhibitors

## Abstract

**Background:**

The pathogenesis and clinical consequences of viral blips are not completely understood. The aim of this large nationwide study was to investigate the incidence of viral blips and factors associated with them, with particular attention to the third (core) agent class—comparing integrase strand transfer inhibitor (INSTI)-based regimens with non-nucleoside reverse transcriptase inhibitor (NNRTI)- and protease inhibitor (PI)-based regimens.

**Methods:**

From the Swedish national InfCareHIV cohort, we included treatment-naïve adults who commenced antiretroviral therapy (ART) from 1 January 2007; data were extracted on 30 September 2020. Viral blips were defined as transient plasma HIV RNA levels between 50 and 500 copies/mL preceded and followed by HIV RNA < 50 copies/mL. Virological failure was defined as two consecutive HIV RNA samples > 200 copies/mL or a single sample of HIV RNA > 1,000 copies/mL, in previously suppressed individuals with more than 12 months since ART initiation. Cox regression was used for event analyses.

**Results:**

In total, 4,210 individuals were included, of whom 853 (20.3%) had 1—6 registered blips. Median observation time was 5.5 years (range 0.5—13.7). Blip incidence was 4.35 (95% CI 4.02—4.69) per 100 person-years. In a sensitivity analysis applying the narrower definition of 50—199 copies/mL, the incidence was 3.80 (95% CI 3.49—4.11) per 100 person-years. Individuals on INSTIs and NNRTIs had similar risk, while those on PIs had a significantly higher risk of blips (hazard ratio [HR] 1.22, 95% CI 1.03—1.45; *p* = 0.02). Transient viremia was not associated with an increased risk of subsequent virological failure; individuals with blips had a lower risk than those with continuously undetectable HIV RNA (HR 0.54, 95% CI 0.41—0.71; *p* < 0.0001).

**Conclusion:**

Individuals on INSTI- and NNRTI-based ART had fewer viral blips than those on PI-based ART. In this cohort, transient viremia below 500 HIV RNA copies/mL was not associated with a higher risk of subsequent virological failure.

**Clinical trial number:**

Not applicable.

**Supplementary information:**

The online version contains supplementary material available at https://doi.org/10.1186/s12879-026-14654-z.

## Background

Viral blips continue to be one of the incompletely understood elements of long-term antiretroviral therapy (ART) in HIV-1 pathogenesis. Even though most researchers and clinicians agree that blips represent short periods (days—weeks) of transient viremia in people with HIV (PWH), there is no widely accepted definition of viral blips regarding maximum HIV RNA level or duration. The current consensus is that blips typically represent a benign phenomenon, although some studies have found an association between blips and an increased risk of virological failure [1–7]. The most widely accepted theory of blip origin is that they are a consequence of temporary activation of viral reservoirs from long-lived virus-producing cells that are not affected by current ART. The released virions are thought to be largely unable to infect and replicate in new cells. This theory is based on previous findings of higher blip frequency in PWH with higher baseline viral loads and later treatment initiation, both of which are associated with a larger viral reservoir [2, 8–10]. The absence of viral evolution further implies that blips are not a consequence of on-going replication [11]. Blips may also reflect measurement inaccuracies in plasma HIV-1 RNA assays, poor adherence, and random variation [2, 11–16]. Studies comparing non-nucleoside reverse transcriptase inhibitor (NNRTI)- or protease inhibitor (PI)-based therapy have shown conflicting results with respect to blip frequency, with reported blip frequencies from 10% to 34% across studies [1, 2, 5, 6, 17–19]. These figures are, however, not directly comparable, as the definition of a blip, the sampling interval and the duration of follow-up differ substantially between studies.

Our aim was to determine the incidence of viral blips in a near-complete national cohort, to compare blip risk between contemporary core agent classes—in particular integrase strand transfer inhibitor (INSTI)-based regimens, for which data remain limited—to describe trends over calendar time, and to assess whether blips predict subsequent virological failure. The study is observational and registry-based and is not designed to resolve the biological origin of blips.

## Methods

Through InfCareHIV, a nationwide database containing > 99% of all clinical real-life data from PWH in Sweden [20], data on eligible individuals from all 30 Swedish clinics treating HIV were extracted on September 30, 2020. We retrospectively included all previously treatment-naïve adults (≥18 years) with HIV in Sweden who started ART from January 1, 2007, when the first INSTI raltegravir was introduced. To ensure virological suppression on ART before follow-up began, individuals were required to have been on ART for ≥6 months and to have at least two HIV RNA samples with <50 copies/mL. ART composition was registered for each plasma RNA sample. Individuals remained in the study as long as their ART consisted of two nucleoside reverse transcriptase inhibitors (NRTIs), either lamivudine or emtricitabine in combination with either abacavir or tenofovir (disoproxil fumarate or alafenamide) together with one core agent: an NNRTI (efavirenz, doravirine, nevirapine, or rilpivirine), a boosted PI (atazanavir, darunavir, or lopinavir) or an INSTI (elvitegravir/cobicistat, bictegravir, dolutegravir, or raltegravir). If an individual had a treatment interruption of <1 month, no samples were analyzed in the following six months. In the case of more prolonged disruption, no more data were registered.

Viral blips were defined as transient plasma HIV RNA levels between 50 and 500 copies/mL preceded and followed by HIV RNA < 50 copies/mL. Measurements less than six weeks apart that otherwise met the criteria were treated as a single blip, for which the highest HIV RNA level was recorded. Transient HIV RNA levels between 20 and 50 copies/mL were registered separately, unless they occurred within six weeks of a blip, in which case they were regarded as the tail of that episode. If the last registered HIV RNA sample was 50—499 copies/mL it was excluded, since it was uncertain whether it represented an emerging blip or secondary virological failure. This definition was chosen for two reasons. First, it is identical to the definition used in our previous study of the same cohort [2], which permits direct comparison of blip incidence between the two study periods. Second, the upper limit of 500 copies/mL was chosen to encompass the range of transient viremia that in Swedish clinical practice does not by itself prompt a change of regimen, while remaining below the single-sample threshold of 1,000 copies/mL used to define virological failure. We note that the upper limit differs substantially between published studies, from 200 to 999 copies/mL [7, 17]. We return to the implications of this in the Discussion.

Individuals were observed from the time point when they reached viral suppression (HIV RNA < 50 copies/mL). Primary virological failure was defined as not reaching this target during the first year of treatment. Secondary virological failure was defined as HIV RNA > 200 copies/mL in two consecutive blood samples or HIV RNA > 1,000 copies/mL in a single sample among previously suppressed individuals, more than 12 months after treatment initiation. Individuals with primary virological failure remained under follow-up, enabling detection of subsequent blips and secondary virological failure. Individuals with secondary virological failure were observed until the event defining the failure occurred and were then excluded.

Self-reported adherence was recorded through the standardized nine-item health questionnaire used routinely in InfCareHIV, which has previously been validated against treatment outcome and satisfaction with care in this cohort [21]. The questionnaire is offered once yearly to all individuals in connection with routine outpatient visits. The adherence item asks how many doses of antiretroviral medication were missed during the preceding seven days, with the response categories none, one to two, and three or more. The item was answered on at least one occasion by 2,584 of 4,210 individuals (61%). As the questionnaire is not administered at every clinical visit, the response proportion is given at the individual level. Because the recall period is seven days, the instrument captures adherence in the week preceding a visit rather than over the interval between visits.

Laboratory test results were collected from clinical data through the InfCareHIV database to which they were sent from all laboratories in Sweden analyzing quantitative HIV-1 RNA. The PCR-methods evolved over the years and included COBAS AmpliPrep/COBAS TaqMan HIV-1 test (version 1.0 and 2.0), COBAS 4800 HIV-1 test, COBAS 6800 HIV-1 test, and the Abbott RealTime HIV-1 assay on the m2000sp/m2000rt platform. Since samples were analyzed at all Swedish virological laboratories over a thirteen-year period and InfCareHIV records the quantified result but not the analytical platform, the specific assay versions in use at each laboratory and time point cannot be retrieved. The lower limit of quantification differed between platforms and decreased over the study period, reaching 20 copies/mL with the assays in use at its end. Demographic and medical data such as age, gender, ethnicity, co-morbidities, biomarkers, laboratory test results, and HIV treatments were available from InfCareHIV.

Genotypic resistance testing was performed by population (Sanger) sequencing of the protease, reverse transcriptase and, where clinically indicated, integrase genes at the virological laboratories, and results were reported to InfCareHIV. Mutations were classified as major resistance-associated mutations according to the Stanford University HIV Drug Resistance Database [22]. Three time windows were analyzed separately: (i) baseline testing, defined as a sample obtained from one year before up to one month after ART initiation; (ii) testing performed within one year after an event of secondary virological failure; and (iii) testing performed within one year after a first viral blip in individuals who did not experience virological failure. For testing after secondary virological failure, mutations were additionally classified as relevant or not relevant to the regimen in use at the time of failure. As all individuals received two NRTIs, NRTI mutations were counted as relevant for all individuals, whereas NNRTI, PI and INSTI mutations were counted only in individuals receiving a regimen of that class. Resistance data are presented descriptively; proportions were compared with the chi-square test.

### Statistical analyses

The time to first viral blip was analyzed with Cox regression with the ART composition as a time-dependent covariate and baseline HIV RNA, CD4+ T-cell nadir, AIDS, and primary HIV infection as covariates; all covariates were entered simultaneously. AIDS was defined as the presence of at least one AIDS-defining condition according to the CDC 1993 classification or the European AIDS surveillance case definition.

The association between transient viremia and subsequent secondary virological failure was analyzed in two separate Cox regressions, one with viral blips and one with episodes of HIV RNA of 20—49 copies/mL as the exposure. In both analyses, the reference group was individuals in whom HIV RNA remained below the limit of detection throughout follow-up.

For each individual, a blip rate was calculated as the number of registered blips divided by the individual’s observation time and expressed per 100 person-years. The incidence reported is the mean of these individual rates, with the corresponding 95% confidence interval. The same approach was applied to the data from our previous study of the same cohort to allow direct comparison between the two study periods.

To assess the influence of the blip definition on the estimated blip incidence, a sensitivity analysis was performed in which the upper limit was lowered to 199 copies/mL, corresponding to the upper limit used in the RESPOND cohort [23], and to 399 copies/mL. Blip incidence was recalculated for each definition using the same approach as for the primary analysis.

The proportion of samples with HIV RNA of 20—49 copies/mL was calculated for each individual, excluding blip samples from the denominator, and the mean proportions were compared between individuals with and without blips using an independent-samples t-test. The proportion of blips among all HIV RNA samples analyzed was compared between calendar years, and the associations between stated adherence and blips, primary virological failure and secondary virological failure were tested, with the chi-square test. Continuous variables, including observation time, the interval between samples and the number of samples per individual, were compared between individuals with and without blips using the Mann–Whitney U test. All *p*-values are two-tailed and considered significant if <0.05. Categorical data are presented as numbers and percentages, whereas continuous data are presented as median with interquartile range (IQR).

**Fig. 1:**
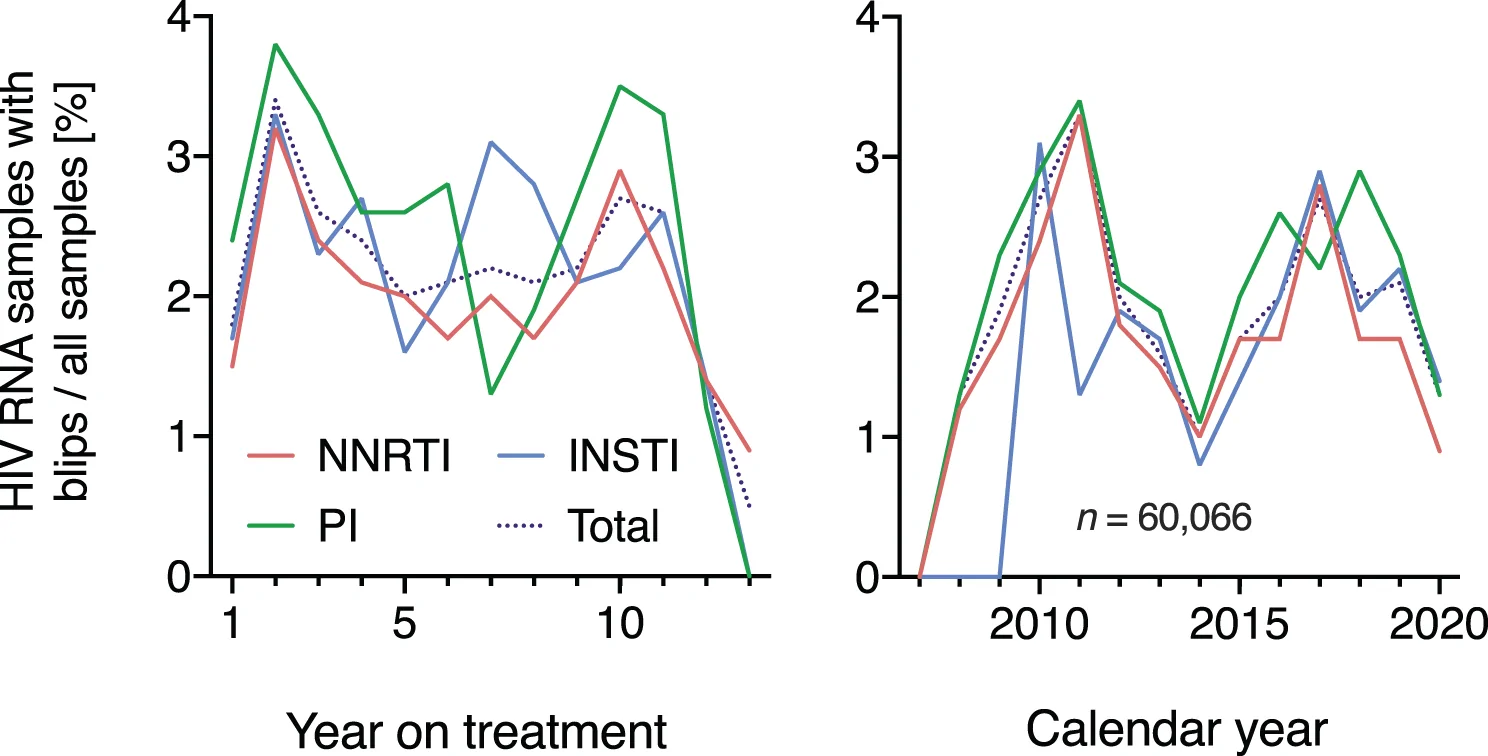
Proportion of HIV RNA samples constituting viral blips, by time on treatment and calendar year. Out of 60,066 HIV RNA samples, 1,180 (2%) constituted transient viremia of 50⎯500 copies/mL. Solid lines represent non-nucleoside reverse transcriptase inhibitor (NNRTI)-based-, protease inhibitor (PI)-based-, and integrase inhibitor (INSTI)-based antiretroviral therapy. All samples are represented by the dotted line. in the left panel, 1 denotes the first year on treatment. The number of samples per calendar year was 420 (2007), 1,192, 2,109, 2,893, 3,437, 3,999, 4,681, 5,330, 5,754, 6,077, 6,646, 6,711, 6,704 and 4,113 (2020); estimates for the earliest years are therefore less precise. The INSTI curve begins in 2009 because raltegravir, introduced in Sweden in 2007, was used only as salvage therapy before the end of 2008. The proportion of blips differed significantly between calendar years (chi-square test for homogeneity, χ^2^ = 109.5, df = 13, *p* < 0.0001)

**Fig. 2:**
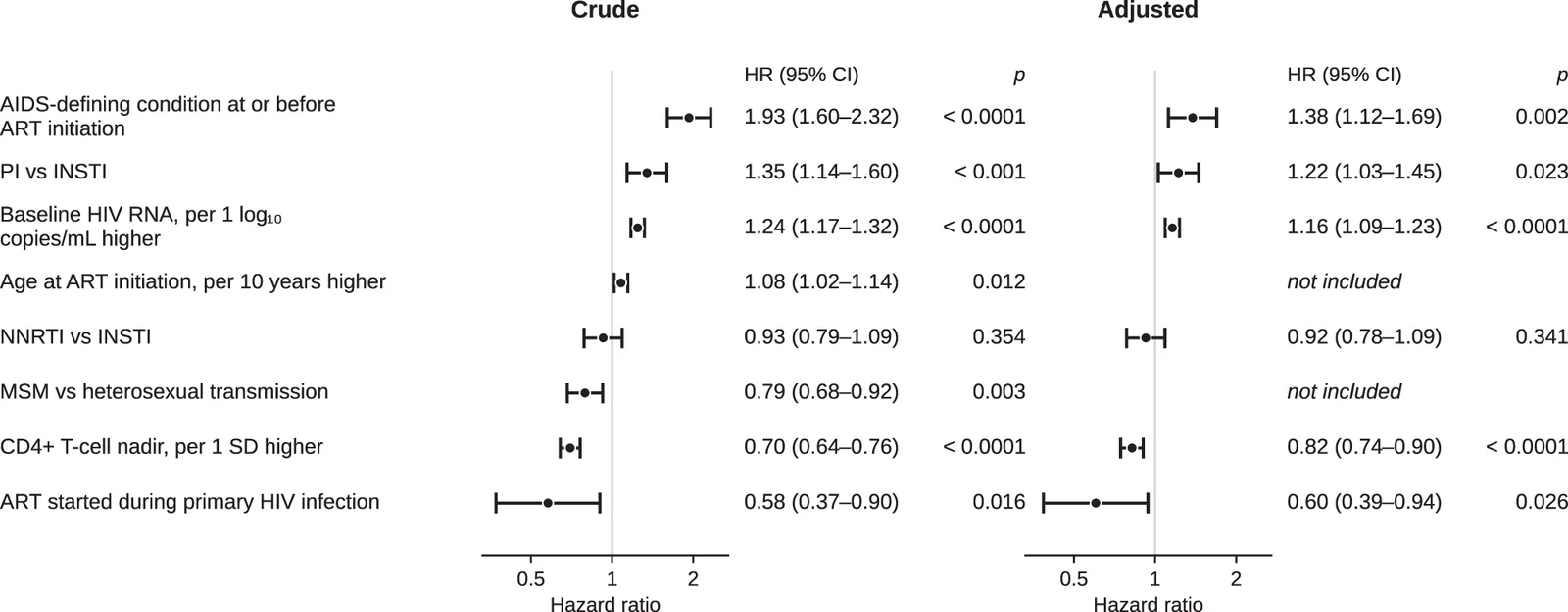
Predictors associated with an increased risk of viral blips. Hazard ratios with 95% confidence intervals estimated by Cox regression in 4,210 individuals, among whom 853 had a first viral blip. The left panel shows crude hazard ratios from univariable models and the right panel hazard ratios from the multivariable model, in which all covariates were entered simultaneously. Hazard ratios above 1 indicate an increased risk of a first viral blip, and the vertical line marks a hazard ratio of 1. Core agent class is compared with INSTI-based regimens as the reference. Age at ART initiation and route of transmission were not included in the multivariable model. ART = antiretroviral therapy, CI = confidence interval, HR = hazard ratio, INSTI = integrase strand transfer inhibitor, MSM = men who have sex with men, NNRTI = non-nucleoside reverse transcriptase inhibitor, PI = protease inhibitor, SD = standard deviation

**Fig. 3:**
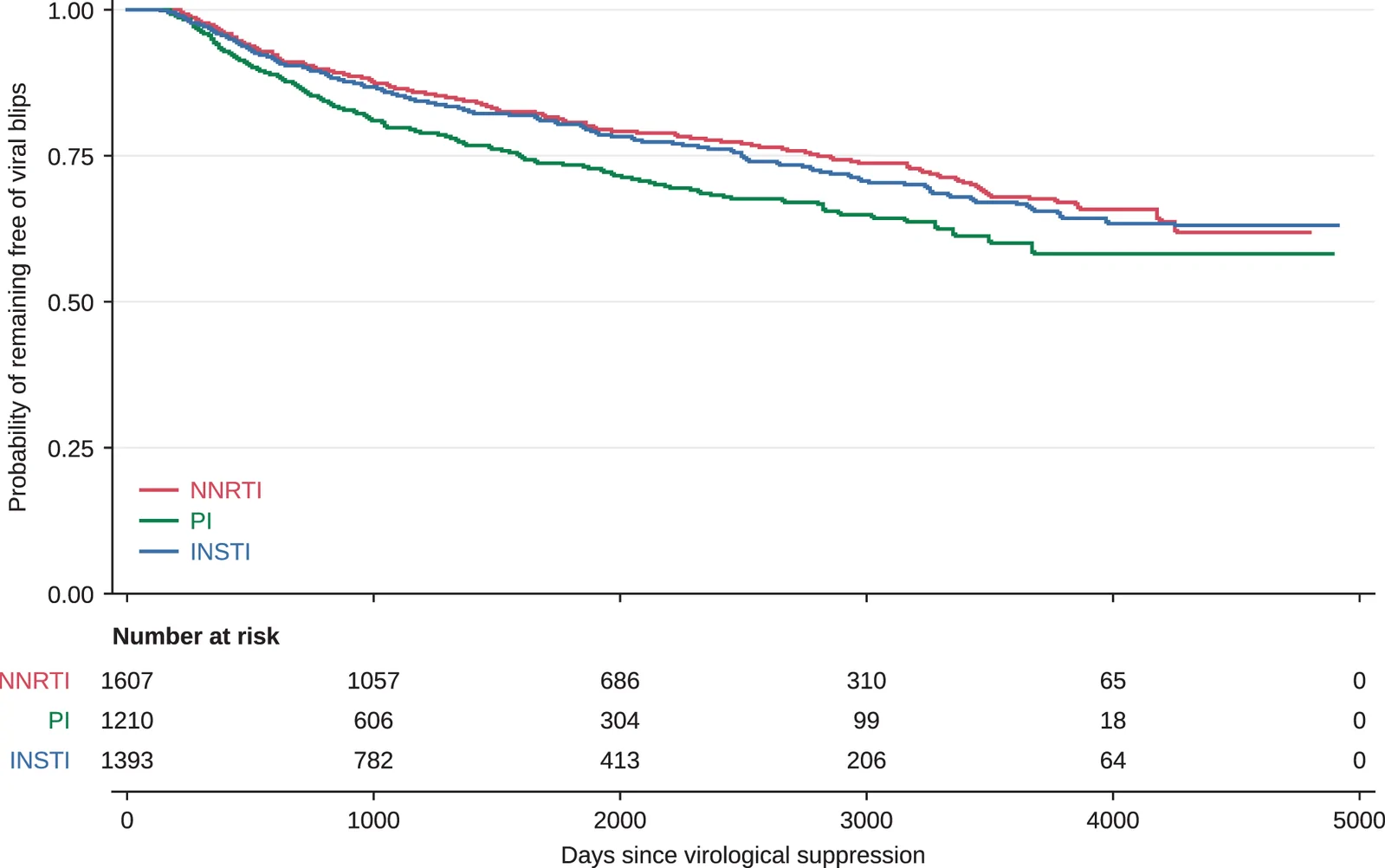
Kaplan-Meier curves for time to a first viral blip, by class of antiretroviral therapy (ART). Time is counted in days from the first HIV RNA measurement below 50 copies/mL. Core agent class was handled as a time-dependent variable, so that follow-up time is attributed to the class an individual was receiving at each point. The three curves differed significantly overall (log rank (Mantel-Cox) test, *p* < 0.0001); in Cox regression, PI-based regimens carried a higher risk of viral blips than INSTI-based regimens (Fig. 2). Numbers at risk are shown below the plot. INSTI = integrase strand transfer inhibitor, PI = protease inhibitor, NNRTI = non-nucleoside reverse transcriptase inhibitor

Cox regression analyses were performed in R version 4.0.1 using the survival package (R Foundation for Statistical Computing, Vienna, Austria). Blip-free survival by ART class was illustrated with Kaplan–Meier curves and compared using the log rank (Mantel–Cox) test. All other statistical analyses were performed using IBM SPSS Statistics for macOS, Version 26.0 (IBM Corp, Armonk, NY). Figure 1 was produced in GraphPad Prism version 8.3 for macOS (GraphPad Software, San Diego, CA) and Figs. 2 and 3 with Matplotlib 3.10 in Python 3.11.

### Use of large language models

A large language model was used to assist with language editing of the manuscript. It was not used to generate or interpret data, and the authors reviewed and approved all resulting text and take full responsibility for its content.

## Results

### Characteristics of individuals with and without viral blips

In total, 4,210 individuals met the inclusion criteria, of whom 853 (20.3%) had verified viral blips with an incidence of 4.35 (95% CI 4.02—4.69) per 100 person-years. Median observation time was 5.5 years (range 0.5—13.7). Individuals with blips had a significantly longer observation period compared with those with continuous viral suppression, median 7.9 years (IQR 5.2—10.6) and 5.0 years (IQR 2.5—7.8), respectively (*p* < 0.0001). Out of 60,066 analyzed blood samples, 1,180 (2%) constituted blips. The median number of registered blood samples per individual was 13 (IQR 7—20), and was higher among individuals with registered blips (Table 1). The median time between each sample was shorter in individuals without blips compared with those with blips, 146 days (IQR 116—174) and 150 days (IQR 130—176), respectively (*p* < 0.05).

**Table 1:** Characteristics of individuals (*n* = 4,210) with respect to viral blips

	Individuals without blips	Individuals with blips
№ of individuals [%]	3,357 (79.7)	853 (20.3)
№ of samples [%]	42,500 (70.8)	17,566 (29.2)
№ samples/individual [median (IQR)]	10 (6⎯16)	17 (12⎯24)
Observation time, years [median (IQR)]	5.0 (2.5—7.8)	7.9 (5.2—10.6)
Age [median (IQR)]	38 (31⎯46)	39 (32⎯48)
Women/men [%]	34/66	34/66
AIDS-defining diagnosis in history [%]	8.8	15.4
HBV positive^1^ [%]	3.9	4.7
HCV positive^2^ [%]	1.9	2.0
Baseline HIV RNA log_10_ copies/mL	4.5 (3.5⎯5.1)	4.9 (4.3⎯5.4)
Nadir CD4+ T-cell count^3^	280 (168⎯410)	227 (120⎯310)
Baseline CD4+ T-cell count^3^	270 (110⎯420)	230 (110⎯330)
Days until first blip	-	947 (589⎯1,739)
№ HIV RNA samples 20⎯49 copies/mL (%)	1,905 (4.5)	1,761 (10.0)
Primary virological failure^4^ [%]	11.7	8.6
Secondary virological failure^4^ [%]	9.6	7.7
Mode of transmission		
Heterosexual [%]	48.0	54.9
Men who have sex with men [%]	35.1	30.8
People with injecting drug use [%]	5.0	3.5
Other^5^ [%]	11.9	10.8

^1^Hepatitis B surface antigen positive

^2^Hepatitis C virus RNA positive

^3^cells/µL, median (IQR)

^4^31 individuals experienced both primary and secondary virological failure

^5^Mother to child, blood products and unknown

HIV RNA samples were drawn more frequently during the first two years on treatment, median four times (IQR 3—5) per year, median two times (IQR 2—3) for the next two years, and median two times (IQR 1—2) for subsequent years.

A majority of individuals were men (66%), and the most common route of transmission was heterosexual. Median age at treatment initiation was 38 years (range 18—86). In individuals with blips, mean number of blips was 1.4 (range 1—6). Median time until the first blip was 947 days (IQR 589—1,739) (Table 1).

The median blip magnitude was 77 copies/mL (IQR 58—117). Of the 1,180 episodes classified as blips, 799 (67.7%) had a peak below 100 copies/mL and 1,044 (88.5%) below 200 copies/mL, while 136 (11.5%), occurring in 129 individuals, were between 200 and 500 copies/mL.

In the sensitivity analysis, restricting the definition to 50—199 copies/mL reduced the number of qualifying episodes to 1,044 and the number of individuals with at least one blip from 853 (20.3%) to 769 (18.3%), with a blip incidence of 3.80 (95% CI 3.49—4.11) per 100 person-years compared with 4.35 (95% CI 4.02—4.69) for the primary definition. With an upper limit of 399 copies/mL the incidence was 4.20 (95% CI 3.87—4.52) per 100 person-years. The mean number of blips per affected individual was essentially unchanged (1.38 and 1.36 for the primary and the narrower definition, respectively).

Quantifiable HIV RNA of 20—50 copies/mL constituted 6.1% of all blood samples (*n* = 3,666). It was significantly more common in individuals with viral blips than in individuals without blips, both as the mean of the proportions calculated for each individual (10.8% versus 4.0%; *p* < 0.0001) and as the proportion of all samples in each group (10.0% versus 4.5%; Table 1). In total, 72% and 32% of all individuals with and without blips, respectively, had episodes with HIV RNA levels between 20 and 50 copies/mL. These episodes were not classified as blips.

Adherence data were available for 2,584 of 4,210 individuals (61%) and acquired through a questionnaire [21]. Among respondents, 89% stated that they took their medication as prescribed, 10% reported having missed 1—2 doses, and 1% three or more doses during the preceding week. There were no associations between stated adherence and blips, or primary or secondary virological failure.

In Fig. 2, the hazard ratios (HR) of predictors that were significant in univariable analysis are shown in a forest plot. In multivariable analyses, a higher risk was found among individuals with AIDS at the time of diagnosis or treatment start (HR 1.37, 95% CI 1.12—1.69; *p* = 0.002) and among those with higher baseline viral load (HR 1.15, 95% CI 1.09—1.23; *p* < 0.0001), while a lower risk was seen in individuals with higher CD4+ T-cell nadir (HR 0.82, 95% CI 0.75—0.90; *p* < 0.0001) and in those starting ART during primary HIV infection (HR 0.60; 95% CI 0.39—0.94; *p* = 0.03). There was no significantly increased risk of viral blips associated with gender, route of HIV transmission, ethnicity, or HIV subtype.

### Antiretroviral therapy

Treatment history was available for all individuals, including those who switched to second-line treatment, at the time of blood sampling. Overall, NNRTI-based ART was most common (40.5%), followed by INSTI (32.1%) and PI (27.4%). Dolutegravir was the most used INSTI (80.3%), followed by elvitegravir/cobicistat (9.5%), raltegravir (8.9%), and bictegravir (1.3%). Tenofovir (disoproxil fumarate or alafenamide) was more common than abacavir as an NRTI component (61.3% and 38.7%, respectively) (Table 2).

**Table 2:** Antiretroviral drugs in use at the time of HIV RNA blood sampling (*n* = 60,066 samples)

ART^1^	All samples (n = 60,066)	Samples without blips (*n* = 58,886)	Viral blips (*n* = 1,180)
**NNRTI**^2^**-based ART [%]**	24,316	23,878 (40.5)	438 (37.1)
Nevirapine [%]	894	878 (3.7)	16 (3.7)
Efavirenz [%]	17,733	17,388 (72.8)	345 (78.8)
Rilpivirine [%]	5,643	5,566 (23.3)	77 (17.6)
Doravirine [%]	46	46 (0.2)	0 (0)
**PI**^3^**-based ART [%]**	16,444	16,087 (27.3)	357 (30.3)
Atazanavir [%]	6,278	6,135 (38.1)	143 (40.1)
Lopinavir [%]	2,748	2,677 (16.6)	71 (19.9)
Darunavir [%]	7,418	7,275 (45.2)	143 (40.1)
**INSTI**^4^**-based ART [%]**	19,306	18,921 (32.1)	385 (32.6)
Raltegravir [%]	1,726	1,699 (9.0)	27 (7.0)
Elvitegravir/cobicistat [%]	1,832	1,791 (9.5)	41 (10.6)
Dolutegravir [%]	15,501	15,190 (80.3)	311 (80.8)
Bictegravir [%]	247	241 (1.3)	6 (1.6)
**Tenofovir-based ART**^5^**[%]**	36,818	36,120 (61.3)	698 (59.2)
**Abacavir-based ART**^5^**[%]**	23,257	22,775 (38.7)	482 (40.8)

Values are numbers of HIV RNA samples with percentages in parentheses. The unit of observation is the individual blood sample, not the individual; an individual contributing samples during more than one regimen appears in more than one row. Percentages in bold rows are proportions of all samples in that column and sum to 100 for the three core agent classes, and separately for the two NRTI backbones. Percentages for individual drugs are proportions within their own drug class and sum to 100 within each class

^1^Antiretroviral therapy

^2^Non-Nucleoside Reverse Transcriptase Inhibitor

^3^Protease Inhibitor

^4^Integrase Strand Transfer Inhibitor

^5^Nine samples were recorded with both tenofovir and abacavir, in connection with a change of regimen, and are included in both rows; the two rows therefore sum to nine more than the column total

The core agent at the first registered blip was an NNRTI, INSTI or a PI in 37.6%, 31.3%, and 31.1%, respectively. Seventy-six percent (*n* = 652) of all individuals with blips remained on their initial therapy at the time of their first recorded blip. In individuals with treatment modifications before the first blip, ART was most often changed to INSTIs (62.7%), followed by PIs (38.9%), and NNRTIs (18.4%). These treatment changes were not related to treatment failure but were most likely due to side effects and convenience (complete data not available). The proportion of blips among all HIV RNA samples analyzed varied over time (Fig. 1), ranging from 0% in 2007 to 3.3% in 2011, and differed significantly between calendar years (*p* < 0.0001). The proportion of samples constituting blips also varied with time since ART initiation: 1.8% during the first year, 3.4% during the second and approximately 2% thereafter.

The probability of viral blips with respect to ART was calculated with time-dependent Cox regression to adjust for the fact that many individuals changed therapy during the study period. With INSTI as a reference, individuals with NNRTI-based ART had a similar risk of viral blips, while individuals with PI-based treatment had a higher risk of developing blips (HR 1.34, 95% CI 1.13—1.60; *p* < 0.001). Results remained similar after adjusting for baseline RNA, CD4 nadir, AIDS and primary HIV (HR 1.22, 95% CI 1.03—1.45; *p* = 0.02) (Fig. 2).

The distribution of individual INSTIs was similar among blip and non-blip samples (Table 2), with no apparent difference between them; the numbers of samples on bictegravir (*n* = 247) and raltegravir (*n* = 1,726) were, however, small, and no formal comparison between individual INSTIs was undertaken.

### Virological failure

Primary virological failure was observed in 467 individuals, and secondary virological failure in 387 individuals, of whom 31 had both. Grouped by the core agent class of the initial regimen, secondary virological failure occurred in 145 of 1,738 individuals on NNRTI-based, 187 of 1,222 on PI-based and 55 of 1,250 on INSTI-based regimens (1.18, 2.24 and 1.33 events per 100 person-years, respectively; chi-square, *p* < 0.0001). Individuals with primary virological failure had no increased risk of developing blips compared with those without primary failure. Individuals with transient viremia of 20—500 HIV RNA copies/mL had a decreased risk of secondary virological failure compared with individuals with sustained suppression. The risk was lower both for those with blips (HR 0.54, 95% CI 0.41—0.71; *p* < 0.0001) and for those with detectable viral load between 20 and 50 copies/mL (HR 0.51, 95% CI 0.41—0.64; *p* < 0.0001), compared with individuals whose HIV RNA remained below the assay’s detection limit.

### Drug resistance

Baseline HIV drug resistance testing, performed by Sanger sequencing and sampled within one year before up to one month after treatment initiation, was available for 55.0% of all individuals (55.6% and 52.9% among individuals without and with blips, respectively). Major NRTI resistance-associated mutations were more common among individuals who subsequently had blips than among those who did not (25.5% versus 19.4%, *p* = 0.004). Major NNRTI resistance was present in 7.3% and 8.9% (*p* = 0.28), major PI resistance in 7.3% and 7.3% (*p* = 0.99), and major INSTI resistance in 1.6% and 3.4% (*p* = 0.04), as defined by the Stanford University HIV Drug Resistance Database [22] (Additional file 1). As all individuals were treatment-naïve at the time of baseline testing, these mutations represent transmitted resistance and are reported by drug class, since no regimen had yet been selected.

Overall, 41% of all individuals with secondary virological failure had a new resistance test performed within one year of this event (130 [40.5%] and 30 [45.5%] for individuals without and with blips, respectively). At the time of testing, median HIV RNA was 4.1 (IQR 3.3—4.8) log_10_ copies/mL. Among those tested, major NRTI resistance was found in 40.8% and 60.0% of individuals without and with blips, respectively (*p* = 0.06), major NNRTI resistance in 25.4% and 26.7% (*p* = 0.88), major PI resistance in 13.8% and 6.7% (*p* = 0.28), and major INSTI resistance in 4.6% and 6.7% (*p* = 0.64). When restricted to mutations conferring reduced susceptibility to a drug in the regimen the individual was receiving at the time of failure, at least one such mutation was present in 50.8% (66/130) of individuals without blips and 66.7% (20/30) of those with blips (*p* = 0.12).

Only 30 (3.5%) individuals with blips but without virological failure were tested for drug resistance within one year after their first viral blip. Fifteen of these had an available baseline result for comparison. In one, a new major NNRTI mutation was detected; in one, a major NRTI mutation present at baseline was no longer detected; and in the remaining 13, the resistance pattern was unchanged from baseline. Among the 15 without a baseline result, four had one to three major mutations across drug classes and 11 had none.

## Discussion

In this retrospective study covering the vast majority of treatment-naïve PWH starting different ART regimens in Sweden between 2007 and 2020 we found that individuals receiving INSTI-based regimens had a decreased risk of viral blips compared with those on PI-based regimens and a similar risk compared with those on NNRTI-based regimens. Viral blips in individuals on INSTIs have only been reported in a few studies, where the incidence has ranged between 1 and 57% [23–28]. Results from previous studies on the association between ART composition and blip risk are conflicting. Several studies report that viral blips are more common in individuals on PI-based compared with NNRTI-based ART [2, 28–31], while the opposite has also been demonstrated [5, 18, 19]. A recent large cohort study has additionally demonstrated a lower blip risk with INSTI-based regimens compared with both PIs and NNRTIs [32]. There are also examples where ART composition had no impact on blip incidence [1, 8, 10, 23]. One possible explanation for the difference observed between core agents is confounding by indication. During the earlier part of the study period, boosted PIs were often preferred for individuals in whom adherence was expected to be problematic, on the grounds of their high genetic barrier to resistance [33]; transient viremia during PI-based ART could therefore in part reflect a case-mix with a higher underlying risk of non-adherence rather than an effect of the drug class itself. This consideration applies with much less force to the latter part of the study period, since second-generation INSTIs have a genetic barrier at least comparable to that of boosted PIs, and it does not explain the whole of the observed difference. We were not able to adjust for this potential confounding, as the reasons for regimen selection are not recorded in InfCareHIV, and this remains an important limitation of the comparison. Adverse effects are relatively common among individuals receiving PIs, and this may further contribute to reduced adherence [34]. In addition, the mechanism of action might play a role: both NNRTIs and INSTIs inhibit viral replication in early stages, whereas PIs block the cleavage of polyproteins into functional enzymes. As RNA-based assays cannot distinguish between infectious virions and immature, replication-incompetent particles, it is conceivable—though not demonstrated here—that transient viremia during PI-based ART more often represents non-infectious virus [35].

Following previous studies, our results show that blip incidence is significantly associated with higher baseline viral loads [2, 8, 9, 23, 28]. This supports the current understanding that blips result from intermittent activation of dormant cells in the HIV reservoir, as the size of the reservoir is believed to be larger in individuals with higher baseline viremia [36]. Supporting evidence of this theory is also found in mathematical simulations and from the results of previous studies, where blip incidence decreased if treatment was initiated during the early stages of primary infection to prevent reservoir build-up [8, 10, 37, 38]. This is in accordance with our results, with a significantly lower risk of blips in individuals diagnosed with primary HIV infection who received a more rapid treatment initiation. A similar pattern has recently been demonstrated in a cohort with known seroconversion dates, where the protective effect of early ART on blip risk persisted even with treatment initiation up to 24 months after HIV acquisition [32].

Individuals who subsequently experienced viral blips were more likely to carry major NRTI resistance-associated mutations at baseline. Pre-existing resistance to a component of the regimen could plausibly permit occasional episodes of transient replication, and intermittent viremia due to pre-existing resistance has been proposed previously [11, 39]. An alternative interpretation is that baseline NRTI resistance acts as a marker rather than a cause, reflecting a longer duration of untreated infection and hence a larger viral reservoir, which is also associated with blips. These findings should be interpreted with caution: baseline resistance testing was available for 55% of the cohort, eight comparisons were made and only the NRTI difference at baseline remains significant when this is taken into account, and the blips observed were not associated with an increased risk of subsequent virological failure.

Similar to a previous study, we found that the proportion of blips differed significantly between calendar years [2], ranging from 0% in 2007 to 3.3% in 2011. We do not interpret this variation as periodic. The calendar-year denominator confounds time on treatment: the proportion of samples constituting blips varies systematically with time since ART initiation, from 1.8% during the first year to a maximum of 3.4% during the second and approximately 2% thereafter. As new individuals entered the cohort continuously while earlier entrants accumulated follow-up, the composition of the cohort with respect to time on treatment changed from year to year, and the calendar-year curve therefore reflects that changing composition rather than a secular trend.

Raltegravir, the first INSTI introduced in Sweden in 2007, was initially used as a salvage therapy for individuals with multi-resistant HIV. It was not until the end of 2008 that it was used as a stand-alone core agent and included in this study. This explains why the blip ratio of INSTIs with respect to calendar year is delayed compared with PIs and NNRTIs. In contrast, during the last year of the study, in line with treatment recommendations favouring dolutegravir as first-line treatment [34], INSTIs were registered as core agent during ongoing ART in 61% of all blood samples analyzed. Four different INSTIs were used during the study period. We observed no apparent difference in blip frequency between them, but this observation is descriptive, the number of observations on bictegravir was small, and our study was not powered to detect differences within the class. Cabotegravir was not yet available during the study period.

Compared with our previous study of the same cohort in 2007—2013, using an identical blip definition [2], blip incidence rose from 2.97 (95% CI 2.28—3.66) to 4.35 (95% CI 4.02—4.69) per 100 person-years. We cannot determine the reason for this difference with certainty. The two study periods differ in length of follow-up, and the progressive introduction of HIV RNA quantification platforms with improved precision near the lower limit of quantification may have affected how often a transient episode was reported above rather than below the 50 copies/mL threshold. We do not interpret the difference as a deterioration in treatment efficacy.

Individuals with blips were observed for a longer period and sampled more often compared with those with continuous suppression. As the time between each HIV RNA sample was longer in individuals with blips, the higher number of observations was not the result of increased sampling frequency. A similar pattern was observed in a study by Joya et al., in which individuals with blips also had longer observation periods and more viral load measurements [19].

Studies differ in how transient episodes with HIV RNA between 20 and 50 copies/mL are separated from viral blips, and in the upper limit applied to blips, which ranges from 200 to 999 copies/mL [7, 17, 31]; some treat 20—999 copies/mL as a single entity [8]. The sensitivity analysis indicates that this choice has a limited influence on the estimated incidence: applying the narrower 50—199 copies/mL definition used in the RESPOND cohort reduced blip incidence from 4.35 to 3.80 per 100 person-years, with overlapping confidence intervals, and reduced the proportion of individuals with at least one blip from 20.3% to 18.3%. The magnitude distribution is strongly right-skewed, with two-thirds of all episodes below 100 copies/mL, and the incidence estimate is therefore not materially dependent on the upper limit chosen.

We found that episodes with HIV RNA between 20 and 50 copies/mL were significantly associated with viral blips and that individuals with these findings had a decreased risk of virological failure. Both outcomes are in line with previous studies [30, 31, 40]. Low-level viremia above 50 copies/mL has, by contrast, been associated with an increased risk of subsequent virological failure [7]. Low-level viremia and blips are both transient and occur at random. As this study is based on clinical data, HIV RNA samples could originate from the start, the peak, or the tail of these events. Considering the two together would reduce the influence of the detection variability seen in viral loads close to the lower limit of quantification [16, 41, 42].

Previous studies show conflicting results as to whether blips constitute a risk of subsequent virological failure [9, 17, 18, 43]. As outlined in recently published studies [19, 28], we found that blips were not associated with an increased risk of secondary virological failure. The explanation for these discrepancies is likely multifactorial. It could be the result of differences in populations, ART regimens, adherence levels, or the use of different HIV RNA quantification methods. Most influential, however, is probably the use of different definitions of blips and virological failure. We applied a definition that we hypothesized would better reflect how clinicians interpret these findings in practice. A consequence of excluding individuals after they developed secondary virological failure is, however, that their observation period decreases and, consequently, their chance of developing blips.

Several studies define virological failure as two consecutive HIV RNA > 1,000 copies/mL, in accordance with the WHO treatment guidelines since 2013 [18, 43, 44]. This approach would, in theory, result in fewer cases of virological failure. Combined with blip definitions up to 1,000 copies/mL, it comes as no surprise that these results differ from ours. With its large study population and long observation time, this study supports previous findings that transient viremia up to 500 copies/mL is not associated with a higher risk of secondary virological failure—an observation of clear clinical relevance [17].

The clinical significance of blips may nonetheless vary with their underlying cause, which may partly explain the conflicting results between studies.

The comparison between core agent classes has been made before, most directly by Dijkstra et al. [28]. Our study adds data from a near-complete national cohort (>99% coverage), with 4,210 individuals, 60,066 HIV RNA samples and up to 13.7 years of follow-up. ART exposure was classified for each blood sample, which accommodates the frequent regimen changes in this population.

One limitation is that the study period includes varying treatment guidelines and quantification methods, which might affect the results. In this study, individuals receiving two-drug regimens (2DR), primarily dolutegravir/lamivudine (DTG/3TC), were not included, and we therefore cannot draw any conclusions regarding this treatment strategy. A recent nationwide Swedish study using the same InfCareHIV registry found that switching to DTG/3TC was associated with lower blip incidence than three-drug regimens [45], and a smaller multicenter cohort study found no significant increase in blip risk with 2DR compared with three-drug regimens [46], together supporting the virological durability of modern 2DR combinations.

The study is also limited by its retrospective nature, where we can only observe and not draw any causative conclusions. Further, it is based on clinical data, and since blip ascertainment depends entirely on when samples happen to be drawn, and the median sampling rate in routine care was 2.2 per year, the incidence reported here should be regarded as a lower bound on the true frequency of transient viremia. In a study in which HIV RNA was measured twice weekly for three to four months, blips were detected in 9 of 10 individuals [11]. Sampling was not protocol-driven, and we cannot exclude that differences in sampling frequency between treatment groups contributed to the observed differences in blip incidence.

The adherence data have important limitations. They were self-reported, answered by only 61% of individuals, and the relevant item covers the seven days preceding the visit. Since the median interval between HIV RNA samples was approximately five months, a period of non-adherence occurring between visits and ending before the last week would not be captured. We therefore cannot exclude that a proportion of the episodes classified here as blips in fact represent the tail of an unreported period of interrupted treatment. For the same reason, our observation that stated adherence was not associated with blips should be interpreted as the absence of evidence rather than as evidence of absence.

## Conclusions

Individuals on INSTI- and NNRTI-based ART were less likely than those on PI-based ART to have subsequent blips. In this cohort, viral blips were not associated with a higher risk of virological failure.

## Electronic supplementary material

Below is the link to the electronic supplementary material.

Supplementary Material 1. Additional file 1: Major HIV-1 resistance mutations by drug class. List of the mutations classified as major resistance-associated mutations in each drug class, according to the Stanford University HIV Drug Resistance Database

## Data Availability

The data that support the findings of this study were extracted from InfCareHIV but are not publicly available due to restrictions related to patient privacy and ethical approval (Dnr: 532-11, Human Research Ethics Committee of the Medical Faculty, University of Gothenburg). Data may be available from the corresponding author upon reasonable request and with permission from InfCareHIV.

## Abbreviations

ART: Antiretroviral treatment
CI: Confidence Interval
DTG: Dolutegravir
INSTI: Integrase strand transfer inhibitor
HBV: Hepatitis B virus
HCV: Hepatitis C virus
HR: Hazard ratio
IQR: Interquartile Range
MSM: Men who have sex with men
NNRTI: Non-nucleoside reverse transcriptase inhibitor
NRTI: Nucleoside Reverse Transcriptase Inhibitor
PI: Protease inhibitor
PWH: People with HIV
SD: Standard deviation
2DR: Two-drug regimen
3TC: Lamivudine
