# Supplementary material for: Understanding viral blips in people with HIV on antiretroviral therapy: a nationwide Swedish cohort study of 4,210 individuals
Source: BMC Infect Dis. 2026 Oct 16;26:1835. doi: 10.1186/s12879-026-14654-z (PMC13685077; doi:10.1186/s12879-026-14654-z)
Supplement: Supplementary file 1 [file 12879_2026_14654_MOESM1_ESM.docx]

## Supplementary Table 1. Major HIV-1 Resistance Mutations by Drug Class (Stanford HIVdb)

| Drug class | Major resistance mutations* |
| --- | --- |
| NRTI (Nucleoside Reverse Transcriptase Inhibitors) | M41L, K65R, D67N, T69D/ins, K70R, L74V/I, F115Y, K219Q/E/N/R, T215Y/F/I/S/C/D/V, M184V/I, Q151M |
| PI (Protease Inhibitors) | D30N, V32I, M46I/L, I47V, G48V, I50V/L, I54V/L/M/A/T/S, V82A/C/F/T/S/M/L, I84V/A/C, N88D/S, L90M |
| NNRTI (Non-Nucleoside Reverse Transcriptase Inhibitors) | K100I, K101E/P, K103N/S, V106A/M, Y181C/I/V, Y188L/H/C, G190A/S/E, P225H, L227C/F/H, L230L/H |
| INSTI (Integrase Strand Transfer Inhibitors) | T66A/I/K, E92G/Q, G118R, E138K, G140A/C/S, Y143C/H/R, Q148H/K/R, N155H, R263K |

*Major resistance mutations are defined as non-polymorphic mutations that cause or contribute substantially to phenotypic drug resistance to the respective drug class, according to the Stanford HIV Drug Resistance Database (HIVdb algorithm version 8.9-1. Stanford, CA: Stanford University. <https://hivdb.stanford.edu>).
